# Supplementary material for: Laboratory detection and rotational spectroscopy of trans-HNSO: implications for astronomical observations
Source: Front Chem. 2025 Dec 3;13:1720662. doi: 10.3389/fchem.2025.1720662 (PMC12708892; doi:10.3389/fchem.2025.1720662)
Supplement: Supplementary file 1 [file DataSheet1.pdf]

Supplementary Material

1 SUPPLEMENTARY TABLES AND FIGURES

Table S1. Calculated equilibrium rotational constants obtained at CCSD[T] level of theory and with different basis sets. All values are in MHz. [a]Root Mean Square Error defined as

$$\mathrm{RMSE} = \sqrt{\frac{1}{n}\sum_{i=1}^{n}(\Delta_i)^2}$$

where n corresponds to three principal axes of inertia, and Δ denotes the difference between the experimental and theoretical rotational constants for each axis.

Parameter	cc-pV(T+d)Z	cc-pV(Q+d)Z	cc-pV(5+d)Z	cc-pwCVTZ	cc-pwCVQZ
A_e	50276.193	50450.393	50758.717	50559.954	50758.761
B_e	9918.615	9971.256	10029.164	9945.199	9999.057
C_e	8284.273	8325.721	8374.490	8310.506	8353.488
RMSE[a]	91.36	21.82	199.77	76.23	194.54

1.1 Figures

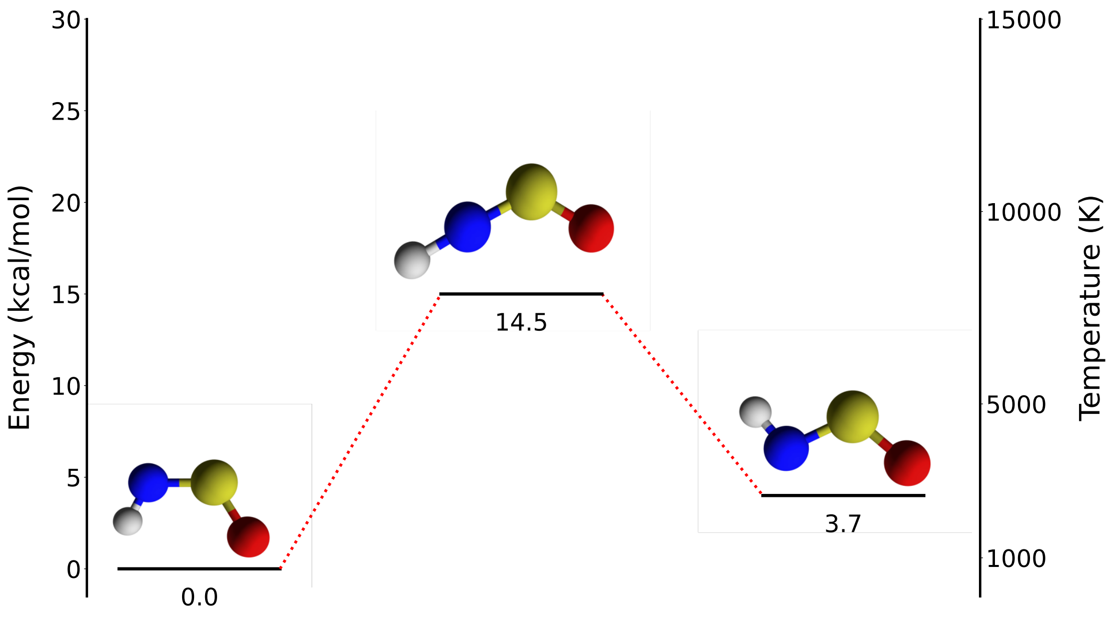

Figure S1. Structures and relative energy levels of *cis*-HNSO (*left*, as the ground state), *trans*-HNSO (*right*, at 3.7 kcal/mol), and the transition state (*middle*, at 14.5 kcal/mol); energy values were derived by **?**. Red atom corresponds to oxygen, yellow to sulfur, blue to nitrogen, and grey to hydrogen.
